# Supplementary material for: Psychometric Properties of the Knowledge of Hydration among Foreign Students of Óbuda University, Hungary
Source: Healthcare (Basel). 2024 Jun 6;12(11):1152. doi: 10.3390/healthcare12111152 (PMC11172119; doi:10.3390/healthcare12111152)
Supplement: Supplementary file 1 [file healthcare-12-01152-s001.zip › healthcare-3016261-supplementary.pdf]

Supplementary Table S1. Item-Total Statistics.

Items	Mean	Std. Deviation	Scale Mean if Item Deleted	Scale Standard Deviation if Item Deleted	Corrected Item-Total Correlation	Squared Multiple Correlation	Cronbach's Alpha if Item Deleted
Do you think drinking water is important to help staying properly hydrated?	0.99	0.07	54.19	11.61	0.05	0.02	0.81
What do you think is the recommended daily Total Water Intake (TWI) (in Litres per day)?	0.41	0.49	54.77	11.49	0.23	0.99	0.80
According to the European Food Safety Authority (EFSA) or World Health Organization (WHO) what do you think is the recommended Total Water Intake (TWI) (in Litres per day)?	0.84	0.99	54.34	11.42	0.15	0.99	0.80
The daily recommended water intake has to come from which of the following?	2.24	1.48	52.94	11.30	0.15	0.99	0.81
According to the European Food Safety Authority (EFSA) or World Health Organization (WHO), the daily recommended Total Water Intake (TWI) has to come from which of the following?	2.85	0.98	52.33	11.43	0.15	0.99	0.80
**Please carefully read each of the following statements about hydration during different moments in your life**							
In an adult, drinking a lot of water is a good way to cleanse the body	8.32	2.25	46.86	9.89	0.71	0.64	0.75
At school and work, adequate hydration is important for proper brain performance and productivity	8.57	2.16	46.62	9.91	0.74	0.72	0.75
During the day everyone should drink at least 2.0 Litre of water	8.07	2.34	47.11	9.84	0.71	0.63	0.75
Staying hydrated can be influenced by some factors (such as Exercise Intensity and Duration, Temperature and Humidity, etc)	8.27	2.24	46.91	9.95	0.69	0.62	0.75
Due to age, the sensation of thirst can be reduced	6.21	2.68	48.97	10.29	0.39	0.19	0.80
Increased water intake contributes to living a healthy life	8.41	2.29	46.77	9.85	0.72	0.67	0.75
